# Women’s perceptions of information about alcohol use during pregnancy: a qualitative study

**DOI:** 10.1186/1471-2458-14-1048

**Published:** 2014-10-08

**Authors:** Amy E Anderson, Alexis J Hure, Frances J Kay-Lambkin, Deborah J Loxton

**Affiliations:** Priority Research Centre for Gender, Health and Ageing, HMRI Building, University Drive, University of Newcastle, Newcastle, NSW 2308 Australia; Priority Research Centre for Translational Neuroscience and Mental Health Research, University Drive, University of Newcastle, Newcastle, NSW 2308 Australia; National Drug and Alcohol Research Centre, University of New South Wales, Randwick, NSW 2032 Australia

**Keywords:** Alcohol drinking, Pregnancy, Information dissemination, Qualitative research

## Abstract

**Background:**

A number of alcohol guidelines worldwide suggest that pregnant women should abstain from alcohol. However, high prevalence rates of alcohol consumption during pregnancy still exist. It is unknown whether there are problems with the dissemination of guideline information that is potentially contributing to such consumption. This qualitative study aimed to explore women’s perceptions of information they received about alcohol use during pregnancy after the introduction of abstinence guidelines.

**Methods:**

Nineteen women from the Australian Longitudinal Study on Women’s Health (ALSWH) 1973–78 cohort that reported a pregnancy in 2009 were recruited for semi-structured telephone interviews. The interviews were conducted until data saturation was reached. Interviews were transcribed, then thematically analysed. ALSWH survey data was used to augment the findings. The main outcome measure was women’s perceptions of information received about alcohol use during pregnancy after the introduction of the 2009 Australian guidelines promoting abstinence during pregnancy.

**Results:**

Women reported a number of problems with the information about alcohol use during pregnancy and with its dissemination. There were inconsistencies in the information about alcohol use during pregnancy and in the advice provided. Mixed messages and confusion about identifying a safe level of consumption had implications on women’s decisions to drink or abstain during pregnancy. Women expressed a need for a clear, consistent message to be provided to women as early as possible. They preferred that the message come from healthcare professionals or another reputable source.

**Conclusions:**

To make an informed decision about alcohol use during pregnancy, women must first be provided with the latest evidence-based information. As this study found a number of limitations with information provision, it is suggested that a systematic approach be adopted by healthcare professionals, in line with best-practice guidelines, to ensure all women are made aware of the alcohol recommendations for pregnancy.

## Background

Alcohol guidelines for pregnancy vary across countries ranging from abstinence to light consumption [1]. Within Australia, these guidelines [2–4] have changed over the past few decades as shown in Table 1. In accordance with other international guidelines, [5–7] the current recommendation is alcohol should be avoided [4]. A similar change occurred in Denmark, when in 2007 guidelines changed from condoning low levels of alcohol use to abstinence [8]. Abstinence is promoted as alcohol is a known teratogen with detrimental effects such as Fetal Alcohol Spectrum Disorders [9, 10]. A safe level of consumption cannot be determined due to inconsistent evidence on the effects of low to moderate alcohol use during pregnancy [11–13].

**Table 1:** **Australian National Health and Medical Research Council alcohol guidelines for pregnancy (1992, 2001, and 2009)**

Year	Guideline
1992	“that abstinence be promoted as desirable in pregnancy” (p. x) [2]
2001	“Women who are pregnant or who may soon become pregnant:
	1. may consider not drinking at all;
	2. most importantly should never become intoxicated;
	3. if they choose to drink, over a week, should have less than seven standard drinks, AND, on any one day, no more than two standard drinks (spread over at least two hours);
	4. should note that the risk is highest in the earlier stages of pregnancy, including the times from conception to the first missed period.” (p. 16) [3]
2009	“For women who are pregnant or planning a pregnancy, not drinking is the safest option.” (p. 5) [4]

Despite recommendations of abstinence, a high proportion of pregnant Australian women still consume alcohol [14]. Previous research found women who drank alcohol prior to pregnancy were more likely to consume alcohol when pregnant during low alcohol guidelines compared to those pregnant during abstinence guidelines [15]. The change in drinking behaviour could be attributable to a change in information pregnant women received, as a Danish study found that after a change from low to no alcohol guidelines, there was an increased proportion (68% to 91%) of general practitioners (GPs) that reported advising all pregnant women about alcohol [8]. It is not clear whether this is the case in Australia.

Little research has examined the information about alcohol use provided to pregnant women. A UK study found that interviewed participants (N = 20) described a lack of clear information and conflicting messages about alcohol use during pregnancy, despite views that a clear recommendation was needed to make informed decisions [16]. They reported that minimal advice about alcohol was provided by their healthcare providers [16]. Limited and inconsistent information about alcohol during pregnancy provided by healthcare providers was also reported by 149 women from 20 focus groups in the US [17]. Australian studies found women were exposed to mixed messages and not always provided with information about the recommendations or potential risks of alcohol use during pregnancy [18–20]. Those studies were conducted prior to the 2009 Australian alcohol guidelines promoting abstinence, so there is a need to explore the information women have received since the introduction of the abstinence recommendation. This can assist in identifying any potential issues with the dissemination of information about the alcohol guidelines for pregnancy. It is worth noting that although the guidelines were released in 2009, a draft version was available in 2007 for public consultation and was advertised by the media and the National Health and Medical Research Council’s website [4]. The purpose of this study was to qualitatively explore Australian women’s perceptions of the information they received about alcohol use during pregnancy after the release of the 2009 abstinence guidelines.

## Methods

### Selection of participants

Participants were sampled from the Australian Longitudinal Study on Women’s Health (ALSWH), which began in 1996 with the recruitment of three age cohorts (i.e. 1973–78, 1946–51 and 1921–26). Women were randomly sampled for the ALSWH from the national health insurance database, Medicare Australia, except women in rural areas were sampled at twice the rate of the representative population in the area. The initial sample for the ALSWH was broadly representative of similarly aged Australian women [21, 22]. Detailed ALSWH recruitment procedures were published previously [21, 22].

For this study, a subsample from the 1973–78 ALSWH cohort was recruited. Women were eligible if they reported being pregnant and had also completed alcohol items in the 2009 survey when the women were aged 31–36 years, or at the 2012 survey when the women were aged 34–39 years. These surveys coincided with the period that the 2009 alcohol recommendations for abstinence during pregnancy were in place. The 2009 survey was sent out on the 31st March 2009, after the abstinence guidelines had been introduced in February 2009. A total of 860 women were eligible for this substudy.

A blinded data manager randomly sampled groups of 10–30 women at a time using a random numbers generator. Five staggered mailouts, which included an invitation letter, information statement and consent form, were sent to 100 women between September 2012 and January 2013. Interested women either mailed back a signed consent form or contacted the researchers by telephone or email expressing a willingness to participate. Telephone calls to participants were made to schedule a date and time for the interview. Interviews were conducted intermittently between October 2012 and May 2013.

After the first 10 interviews had been conducted, sample characteristics were run to assess the sampling technique, which was found to be sufficient in achieving variability amongst participants (e.g. drinkers and abstainers). The random sampling of participants resulted in a sample with diverse characteristics, which allows for representativeness of a topic to be achieved within qualitative studies [23]. Only women who contacted the researchers and consented to participate were included in this substudy. Non-responders were considered to be non-consenters. All consenters had reported pregnancies in the 2009 survey only.

### Data collection and instruments

Women were invited to participate in semi-structured, audio-recorded, telephone interviews. Telephone interviews allowed the researchers to interview women from across Australia, which would not have been possible if face-to-face interviews were chosen due to limited funding. Additionally, telephone interviews have been found to provide a comfortable environment to build rapport and facilitate the disclosure of personal information, resulting in high quality data [24]. Interviews were conducted until data saturation was reached [25]. As the interviews were semi-structured, a list of questions (see the ‘List of questions used to guide the interviews’ section) was used to guide the interviews but was not strictly followed as participants’ experiences varied, which required a flexible approach to be taken during data collection. The length and time of interviews were adapted to accommodate the participants’ schedules.

### List of questions used to guide the interviews

- Can you tell me about your last pregnancy? How was your last pregnancy?
- How did you feel? How was your health?
- What sort of advice or information were you given the last time you were pregnant?
- For example, what was the advice/info you were given about food or exercise?
- Who gave you the advice/info?
- Can you tell me about how that conversation started?
- During your most recent pregnancy what were you told about alcohol use during pregnancy?
- Can you tell me about conversations you might have had with different people about drinking alcohol during pregnancy?
- (if no mention of health care providers) What information did you receive from: your GP? your midwife? your obstetrician?
- How else did you get information about recommended alcohol use for pregnant women?
- Where did you get information? (books, websites etc.?)
- (If they didn’t get any information), Where do you think pregnant women find out about the recommendations for alcohol use during pregnancy?
- What sort of information/advice did you get/receive/find? What did you think about the information?
- How did the information affect your decision about what you would do during pregnancy?
- What sorts of other information or advice have you heard of other pregnant women receiving?
- And what do you think about that? How did they get that information?
- What other things would you like to say about drinking alcohol during pregnancy?
- Could you please tell me more about that? Or could you please elaborate on that?

To ensure consistency in data collection, only one researcher [AA] conducted all interviews, which were carried out in a specified telephone interview room. Notes were taken during the interviews, and a logbook was used after the interview to allow the interviewer to reflect on what was said. The female interviewer was a PhD student, who had been trained in qualitative techniques during her Bachelor of Psychology degree and through additional qualitative courses offered by the Australian Consortium for Social and Political Research.

Participant characteristics during their 2009 pregnancies were derived from the ALSWH 2009 survey. The items from the ALSWH survey that were used to describe participants included sociodemographic characteristics and health behaviours as seen in Table 2. To reduce the potential for bias, the interviewer was blinded to participants’ survey data until after each interview. Interview data were linked with the survey data, which allowed the researcher to avoid asking about participants’ alcohol consumption during pregnancy or questions that were repetitive.

**Table 2:** **Interview participants’ socio-demographic and health behaviour characteristics during pregnancy (N = 19)**

Characteristics at time of pregnancy (2009)	n (%)
Marital status	
Married	17 (89.5)
De facto	2 (10.5)
Number of children	
0	8 (42.1)
1	8 (42.1)
2	3 (15.8)
Rurality	
Major cities	10 (52.6)
Inner regional	4 (21.1)
Outer regional	3 (15.8)
Remote	2 (10.5)
Employment	
No paid work	2 (10.5)
Part-time work (1–24 hours/week)	8 (42.1)
Full-time work (35-49+ hours/week)	9 (47.4)
Highest level of education	
Year 12 or equivalent	2 (10.5)
Certificate/diploma	5 (26.3)
University degree	9 (47.4)
Higher university degree	3 (15.8)
Household annual income	
No income	1 (5.3)
$36,400 - $51,999	2 (10.5)
$78,000 - $103,999	5 (26.3)
$104,000 - $129,999	1 (5.3)
$130,000 - $155,999	3 (15.8)
$156,000 or more	7 (36.8)
Health Care Card (covers healthcare costs for government concession recipients)	
No	17 (89.5)
Yes	2 (10.5)
Private health insurance	
No	4 (21.1)
Yes	15 (78.9)
Smoking status	
Never smoker	12 (63.2)
Ex-smoker	6 (31.6)
Smoker > =20 per day	1 (5.3)
Illicit drug use (ever)	
Never used illicit drugs	8 (42.1)
Used illicit drugs	11 (57.9)
Change in alcohol intake from before pregnancy to during pregnancy	
Non drinker	3 (15.8)
Drinker to abstainer	4 (21.1)
Drinker decreased intake (i.e. decreased usual frequency and/or quantity)	10 (52.6)
Drinker same intake	1 (5.3)
Unknown due to missing data	1 (5.3)
Frequency of alcohol use during pregnancy	
Did not drink alcohol	7 (36.8)
Less than once a month	6 (31.6)
Less than once a week	2 (10.5)
1 or 2 days per week	3 (15.8)
3 or 4 days per week	1 (5.3)
Quantity of alcohol use during pregnancy	
Did not drink alcohol	7 (36.8)
1 or 2 drinks per day	12 (63.2)

### Ethical considerations

The ALSWH was granted ethical clearance by the Universities of Newcastle and Queensland (Ethics approvals H0760795 and 2004000224) on the 26th July 1995. Ethics clearance for this substudy including ALSWH participants was provided on the 2nd May 2012 by the ALSWH Publications, Substudies and Analyses Committee (project #W085) and on the 4th July 2012 by the University of Newcastle (Ethics approval H-2012-0153). Participants provided written or verbal informed consent, and were given an opportunity to ask questions at the beginning and end of the interview. They were informed that they could stop the interview or withdraw from the study at any time. It was made clear to participants that all data would be reported in a de-identified manner. Although it was not expected that the interviews would cause any distress, there were procedures in place to refer women to support services if they became distraught during the interviews.

### Data analysis

Descriptive statistics were conducted in SPSS (version 19) using the 2009 survey reporting participant characteristics and alcohol intake during pregnancy. Data measuring the usual frequency and quantity of alcohol use from 2006 and 2009 were used to examine changes in drinking behaviour from before pregnancy to during pregnancy.

Coming from a realist perspective, the interviewer decided to take a pragmatic approach to analysing the data [26, 27]. Interviews were transcribed primarily by a transcription company and checked by the interviewer [AA]. Data were managed using NVivo 10 [28]. Transcripts were thematically analysed by one coder [AA]. Thematic analysis was chosen as it has been described as a flexible and pragmatic analytic technique, rather than being strictly defined by a particular theory or epistemology [29]. A semantic level thematic analysis, focussing on the surface meanings of the data, was utilised to answer the research question [29]. Due to the variability in participant characteristics, particularly with respect to drinking behaviour during pregnancy, a wide range of views was gathered and led to data saturation. Data saturation was reached when the information from interviews became repetitive and no new relevant information emerged [25].

The coder used Braun and Clarke’s guide for thematic analysis, involving: familiarisation with the data; initial code generation; developing potential themes; reviewing themes with extracted data; clearly defining themes; and extracting data to utilise as thematic examples in the manuscript [29]. The coder familiarised herself with the data by having conducted the interviews, reviewing the transcripts after transcription, and reading the transcripts multiple times before and during coding. The coder read through transcripts sequentially and assigned codes to selections of text. The coder kept a logbook during the coding process to describe the creation of themes from grouping of the codes. Themes were generated inductively. As the analysis continued, potential themes were refined. A thematic skeleton was created to assess the themes in relation to the relevant codes and quotes from the transcripts. Themes were defined, and quotes that reflected the varying experiences and meanings from the data were chosen for the manuscript. Throughout the analysis, the coder was supervised by the senior investigator [DL], which involved meeting multiple times to review and discuss the coding and thematic structures throughout the analytic process. Data were constantly reviewed to ensure themes reflected participants’ narratives. The RATS guidelines were used to make sure the manuscript adhered to quality reporting of a qualitative study [30].

## Results

Nineteen women (19% of those approached) were interviewed. An additional two women mailed back signed consent forms, but were unable to be contacted for interviews after multiple attempts. None of the 81 non-participants (81% of those approached) explicitly opted out of the study by actively declining participation. Interviews lasted an average of 46 minutes, ranging from 20 to 78 minutes.

Socio-demographic and health behaviour characteristics for participants are included in Table 2. Participants were aged 31–36 years (M = 33.73, SD = 1.77) when pregnant in 2009. At the 2009 survey, around half of the women were from major cities, worked full time and had a university degree. During their 2009 pregnancies, 42% of the women were pregnant with their first child, whereas the remaining 58% already had at least one child. Most women altered their drinking behaviour from before pregnancy to during pregnancy. Twelve women reported drinking alcohol during pregnancy (63%) and seven abstained (37%). Of the twelve women who consumed alcohol during pregnancy, the majority (67%) drank less than once a week and none of them usually drank more than 1 or 2 drinks on a drinking day.

### Themes

#### A faulty information delivery system

It was apparent from the outset of the analysis that no consistent message about alcohol use was systematically provided to pregnant women. On the contrary, there were multiple messages from a number of different information sources. This overarching theme encompassed a number of subthemes describing faults in the information pool and pathways. Differences were seen between the amount of information obtained, the recommendations about alcohol use during pregnancy, and the interpretation of the recommendations.

### Information overload versus no information

Most of the women described the amount of overall information provided during pregnancy as overwhelming, particularly with their first child. Being overwhelmed had consequences for women’s ability to process the information, as one woman mentioned, ‘*I disregarded a lot of the advice because I felt overwhelmed’ (Participant 11).* The women were given a range of information (e.g. healthcare choices, healthy lifestyle factors) by a number of sources, such as books, media, formal education, healthcare providers, family, friends, websites, and antenatal classes. Those who found conflicting information between sources, would sometimes create a hierarchy, often relying on healthcare providers to explain the discrepancies and as one woman mentioned, to *‘just steer me in the right direction’ (Participant 15)*.

Not all women were overwhelmed, with one woman feeling more comfortable with the more information she got. Other women described a lack of information, particularly on lifestyle factors such as alcohol use. Self-sourcing information in the absence of it being provided was common, as one woman put it, ‘*GP gave me nothing, obstetrician gave me nothing… it's all about the pregnant me sourcing it’ (Participant 5).*

Women differed in the amount of information they received about alcohol use during pregnancy, with some getting recommendations from a number of sources and others not getting told anything. Some women were provided with information by healthcare providers, but generally not prior to or at pregnancy confirmation, but rather weeks later at their first antenatal appointment closer to their second trimester. Those who were not advised by a healthcare provider believed it was because they were non-drinkers or did not ‘*look like someone that would be swigging away at some alcohol every night’ (Participant 6)*. Many women did not receive as much information in subsequent pregnancies compared with their first. Not receiving information had implications for how they then made their decisions about whether or not to drink during pregnancy:

*I don't remember getting any formal information, but I think I just had in my head that, you know, healthy lifestyle is important, so I sort of ate well and sort of didn't have three or four drinks if I went out for dinner or something. I'd only have one or two, sort of take a bit more care of my health. I couldn't say where I got the reasoning for that. I think that's just a build-up of information over my lifetime sort of thing. (Participant 9)*

*It’s [alcohol advice for pregnancy] not promoted anywhere. To me, that’s a bit of a concern for me, that women perhaps just aren’t getting the advice. At least, if… you’ve got the advice and you’ve got the information, you can make the decision. (Participant 10)*

### What is the recommendation anyways? Depends who you ask

It was common knowledge that heavy alcohol use was not recommended during pregnancy, and that alcohol should be avoided during the first trimester. However, there were discrepancies in the recommendation that women received about a safe level of consumption, varying from abstinence to light consumption:

*I have this really vivid image of, during my first pregnancy,… [the GP] saying that it’s now recommended that you don’t have any alcohol… in the second one I’m sure that was reiterated. (Participant 16)*

*He [my obstetrician] did say that it's not ideal, but the odd glass here and there wouldn't hurt. (Participant 17)*

Some women were aware that recommendations had changed over time, believing this reduced the strength of the message. When faced with this inconsistency, women sometimes relied on personal experience or the experience of others to determine which message they chose to believe:

*They'll say small amounts of alcohol are okay. Then we go back to saying no alcohol during the pregnancy. Women kind of think well hang on, I've got lots of friends that did drink small amounts of alcohol during their pregnancy and their kids seem fine. So they don't place as much importance on that. (Participant 4)*

Other messages regarding alcohol in general or other pregnancy issues often clouded the message about alcohol use in pregnancy. Some women heard alcohol, particularly wine, was beneficial because it contained antioxidants, promoted better sleep, and reduced stress. One participant believed stress was more hazardous during pregnancy than drinking alcohol, so she thought it was fine to have a glass of wine occasionally. Alternatively, another woman could not see any benefits in consuming alcohol during pregnancy.

### Interpreting a grey area: ‘no safe level’ versus ‘no harm shown’

A number of women discussed how information defining a safe level of alcohol use was mixed. Some women expressed confusion or frustration about this, with one woman stating, *‘I just can’t see why there is that grey area’ (Participant 3)*. She could not understand why the information was unclear because there was no reported benefit of drinking during pregnancy. Another woman believed a grey area meant the evidence was not strong enough to support a recommendation of abstinence:

*If it was that it was absolutely detrimental and more than one glass could kill the baby… and you had scientific evidence to back that up, well then that's the message that should be communicated… But I think it's such a grey area. (Participant 17)*

Some of the women with science or health backgrounds understood the evidence for a safe level of consumption is inconclusive. This grey area led to two main interpretations. A number of women believed in a better safe than sorry approach, such as ‘*If you don't know what the result is, don't do it. It's as simple as that’ (Participant 2).* Whereas, other women had a relaxed approach, reflected by one woman saying, ‘*There is no research to suggest that a couple of drinks is okay or not… to me that means that it's okay to have one or two now and then’ (Participant 7).*

#### Improving the information delivery system

It became apparent during interviews that women had opinions on how to address faults in the information delivery system. This second overarching theme was therefore derived through further exploration of the first theme. Women believed a clear, consistent message needed to be delivered early on by a reliable source, as described in the three following subthemes.

### Clear, consistent, and strong recommendation

Women believed the recommendation needed to remain consistent over time and be clearly delivered. Women who thought the recommendation should be abstinence and those thinking it should be low alcohol intake both believed that one message should be chosen and continued:

*Stick with that message and keep that message going for years, not just, okay, this week it's that message and next week it's another. I think that's where people lose face… I think being consistent is really the only way to continually get a message across. (Participant 8)*

One woman did not think a single message was possible, believing recommendations should be based on the individual. Although other women believed individual differences were relevant, they still thought a clear message was needed. One reason for this was to avoid individual interpretations, such as if the message was abstinence then some women might decide one drink was safe, but if it was one drink was okay then they may decide two drinks was alright. A straightforward message of abstinence was suggested as a way of dealing with individual differences.

A number of women believed the message needed to be strong, with some suggesting scare tactics to make it more tangible. Women educated about Fetal Alcohol Spectrum Disorders thought visual depictions of children affected with these disorders could shock women into abstaining. Other women believed scaring pregnant women could cause undue stress, which could be harmful for the woman and fetus. Generally women thought the message would have more impact if reasons for the message were included:

*People need to be made aware of the effects of drinking alcohol during pregnancy… People aren't just going to take it on face value. They need to know, well what's going to happen if I do have it. (Participant 4)*

### A reliable source with a vast reach

The strength of the message was also thought to be influenced by the source of information. Women viewed healthcare providers as reliable sources with expert knowledge. A hierarchy among healthcare providers was described, but this varied depending on the type of care received. A number of women thought doctors, primarily obstetricians, were more knowledgeable then nurses and midwives, but other women thought midwives knew more than doctors. Despite these discrepancies, most women believed the alcohol message should be provided by healthcare providers:

*The only cohesive factor in all that is the person that's giving you the [health]care while you're pregnant. Because not all women will read books, not all women have access to the internet… or use the internet. (Participant 5)*

Additionally, women mentioned a need to utilise sources such as television, printed media, social media and websites to raise awareness of the current recommendations, since they have changed over time. Such an approach was said to help ‘*get rid of that old thinking’ (Participant 9)* from previous pregnancies, which may be outdated. Some women expressed a need to target certain groups to ensure all women within Australian society are aware of the alcohol recommendations for pregnant women. One woman said information needed to be provided *‘in a lot of different locations that people of all classes can access’ (Participant 15).* Regardless of how they thought the message should be delivered, women believed it should come from a reputable source to have an impact. In addition to healthcare providers and healthcare bodies, the government and universities were considered valid sources for passing on alcohol recommendations to pregnant women.

### Early information provision

Women believed advice about alcohol recommendations should be provided before the first antenatal appointment, which was often late in the first trimester or the beginning of the second trimester. They were aware that the first trimester is a crucial time for development, so information was wanted early:

*Your first 12 weeks, as you know, it's the most critical… so you want to get it[information]… before that time. It's a bit late when you go to your doctor for your eight week, 10 week scan. (Participant 2)*

Women suggested information be provided when planning a pregnancy or at the GP when getting a pregnancy confirmed. The women acknowledged that not all pregnancies are planned, so they considered the GP visit for pregnancy confirmation a critical teachable moment:

*That's [the GP visit for pregnancy confirmation] when you're taking in the most information… You're trying to learn everything. I think that's where you need to really nail it and get the message across. (Participant 6)*

Some women thought information about alcohol use in pregnancy should be part of education in schools. The women thought it may deter students from having unprotected sex while drinking alcohol, as well as making it common knowledge from a young age.

## Discussion

### Main findings

This is the first study to investigate women’s perceptions of information they received about alcohol use during pregnancy after the Australian alcohol guidelines were changed from low drinking to abstinence in 2009. This bottom-up approach provided an understanding of how alcohol guidelines have filtered down to pregnant women. Gaps within the information pathways were identified, as were potential solutions to address these gaps. It was apparent that for these women a number of inconsistencies existed within the information delivery system in relation to alcohol use during pregnancy. There was a lack of clarity in the available evidence and the advice provided, which in turn impacted the ways in which women interpreted the recommendations about alcohol use during pregnancy. Women expressed that a clear message about alcohol use and pregnancy needed to be maintained over time and delivered early in pregnancy from a reputable source.

### Interpretation

Healthcare providers were believed to be an ideal source of information. This finding coincides with an Australian survey that found over 90% of women believed healthcare providers should assess alcohol use in pregnancy, provide information about the harms of antenatal alcohol consumption and advise abstinence [31]. Internationally, studies have found most women want a clear message about alcohol use in pregnancy from healthcare providers [16, 17, 32]. Women in this study believed doctors should know the latest research and would advise accordingly. This is worrisome considering variations that have been reported in the levels of knowledge and behaviours of healthcare providers with regards to recommendations for alcohol consumption during pregnancy [8, 33–36]. For example, within Australia less than half of healthcare providers routinely assessed alcohol use during pregnancy, and less than a third routinely provided information about the harms of antenatal alcohol use [36, 37]. It is not surprising than to find variation among the women in this study with regards to the information or advice they received from healthcare providers. Improved translational efforts between policy makers, researchers and healthcare providers need to occur, along with clarification about when alcohol use screening and recommendations should be provided and by whom.

Women in this study believed information about alcohol and pregnancy was needed early, however this did not occur for many of them. Early information provision is important because, although the teratogenic effects of alcohol can occur at any time, there is an increased risk during the first trimester [38, 39]. Even guidelines that condone light drinking in later pregnancy recommend abstinence in the first trimester [40]. To provide information early, the primary care sector needs to be involved. GPs are usually the first healthcare providers that pregnant women have contact with, either to discuss planning a pregnancy or confirming a pregnancy. However, around half of pregnancies are unplanned, potentially increasing the risk of alcohol exposure during a critical phase of development [41, 42]. Clinical guidelines recommend that GPs assess alcohol use and advise about potential adverse effects during pregnancy not only when treating pregnant women or those planning a pregnancy, but also when talking with women of child-bearing age who may become pregnant [43, 44]. Multifaceted strategies aimed at increasing GPs’ adherence to these guidelines should be considered, as strategies targeting multiple levels (e.g. individuals, organisations, and society) are likely to be more effective than a single approach [45].

To assist healthcare providers in advising women, and to satisfy women’s requests for consistency expressed in this study and others, [16, 17] the recommendations about alcohol use in pregnancy should be maintained over time. Variations in recommendations caused confusion among women and were seen as lacking credibility. These findings coupled with previous research that found women were less likely to consume alcohol under abstinence guidelines [15] suggests that the current recommendations should be upheld. Mass media campaigns could help raise awareness of the official recommendations. These alternative strategies, particularly that target the broader population, are critical given that in the face of conflicting messages about alcohol, women in this study and others [16] relied on their previous pregnancy experiences or that of others to determine a safe level of consumption during pregnancy. This is problematic considering recommendations can change between pregnancies and a number of women received little or no information during subsequent pregnancies. Consistent information provision regardless of prior pregnancy experience is needed to ensure equal access to the latest evidence-based information.

### Strengths and limitations

This study contained a small sample which may be considered as a limitation by some readers. However, not only was data saturation reached, but the random sampling technique ensured that a variety of women were represented in the study, particularly both drinkers and abstainers during pregnancy. Such variability ensured that a variety of perceptions was achieved. Consenters were not compared to non-consenters, as the latter did not provide consent for their survey data to be included in this substudy. Although the qualitative design of this study means that findings are not meant to be generalisable, a number of results from this study were consist with those of international qualitative [16–20] and quantitative studies [31, 32]. Consistencies with previous research combined with the diversity among study participants suggest conceptual generalisability was most likely achieved. In addition, trustworthiness was also demonstrated by creating transparency throughout each stage of the research process and keeping an ‘audit trail’ so that the study could be subject to external scrutiny. Women who frequently consume heavy amounts of alcohol during pregnancy were not represented in this study, as participants reported having no more than two drinks on a drinking day. Although no formal inter-rater reliability measure was applied, the coder discussed and reviewed the coding process and structure with the senior investigator. Additionally, the existing qualitative and quantitative literature on this topic was used to provide additional context when interpreting results. There was a short timeframe between the 2009 alcohol guidelines being introduced (February 2009) and the measurement of women’s pregnancies through the ALSWH survey (mailed out 31st March 2009). However, the draft guidelines were available as early as 2007 and a media release promoting the new guidelines was sent out before the ALSWH survey had been mailed out. Regardless of how the guidelines were disseminated, they were the current guidelines at the time of the women’s pregnancies.

## Conclusion

The discord between women’s expectations to receive information about alcohol use early in pregnancy from their healthcare providers and the lack of consistent information actually being provided could be addressed by introducing a multifaceted, systematic approach to information delivery. Such an approach, particularly within the primary care setting, could help ensure a clear and consistent message is sent through this information channel which women believe to be a reliable source. Alcohol recommendations should be maintained over time to provide a stable platform for this information provision to occur. Providing women with evidence-based information will enable them to make informed decisions about drinking during pregnancy.
